# Preventive treatment with ginsenoside Rb1 ameliorates monocrotaline-induced pulmonary arterial hypertension in rats and involves store-operated calcium entry inhibition

**DOI:** 10.1080/13880209.2020.1831026

**Published:** 2020-10-23

**Authors:** Rui-Xing Wang, Rui-Lan He, Hai-Xia Jiao, Run-Tian Zhang, Jing-Yi Guo, Xiao-Ru Liu, Long-Xin Gui, Mo-Jun Lin, Zhi-Juan Wu

**Affiliations:** a The Key Laboratory of Fujian Province Universities on Ion Channel and Signal Transduction in Cardiovascular Diseases, The School of Basic Medical Sciences, Fujian Medical University, Fuzhou, People’s Republic of China; b Department of Physiology and Pathophysiology, The School of Basic Medical Sciences, Fujian Medical University, Fuzhou, People’s Republic of China; c Department of Biochemistry and Molecular biology, The School of Basic Medical Sciences, Fujian Medical University, Fuzhou, People’s Republic of China

**Keywords:** Pulmonary arterial smooth muscle cells, pulmonary arterial pressure, pulmonary vascular remodelling, right ventricle hypertrophy

## Abstract

**Context:**

Ginsenoside Rb1, the main active ingredient of ginseng, exhibits *ex vivo* depression of store-operated calcium entry (SOCE) and related vasoconstriction in pulmonary arteries derived from pulmonary hypertension (PH) rats. However, the *in vivo* effects of ginsenoside Rb1 on PH remain unclear.

**Objective:**

This study explored the possibility of using ginsenoside Rb1 as an *in vivo* preventive medication for type I PH, i.e., pulmonary arterial hypertension (PAH), and potential mechanisms involving SOCE.

**Materials and methods:**

Male Sprague-Dawley rats (170–180 g) were randomly divided into Control, MCT, and MCT + Rb1 groups (*n* = 20). Control rats received only saline injection. Rats in the MCT + Rb1 and MCT groups were intraperitoneally administered single doses of 50 mg/kg monocrotaline (MCT) combined with 30 mg/kg/day ginsenoside Rb1 or equivalent volumes of saline for 21 consecutive days. Subsequently, comprehensive parameters related to SOCE, vascular tone, histological changes and hemodynamics were measured.

**Results:**

Ginsenoside Rb1 reduced MCT-induced STIM1, TRPC1, and TRPC4 expression by 35.00, 31.96, and 32.24%, respectively, at the protein level. SOCE-related calcium entry and pulmonary artery contraction decreased by 162.6 nM and 71.72%. The mean pulmonary artery pressure, right ventricle systolic pressure, and right ventricular mass index decreased by 19.5 mmHg, 21.6 mmHg, and 39.50%. The wall thickness/radius ratios decreased by 14.67 and 17.65%, and the lumen area/total area ratios increased by 18.55 and 15.60% in intrapulmonary vessels with 51–100 and 101–150 μm o.d.

**Conclusion:**

Ginsenoside Rb1, a promising candidate for PH prevention, inhibited SOCE and related pulmonary vasoconstriction, and relieved MCT-induced PAH in rats.

## Introduction

Pulmonary hypertension (PH), characterized by increased pulmonary vascular tone, vascular smooth muscle hyperplasia, and remodelling in distal pulmonary arteries, is a severe condition characterized by elevated pulmonary arterial pressure (PAP) that eventually leads to right heart failure and death (Huetsch et al. 2016; Simonneau et al. 2019). PH is divided into five categories according to cause, i.e., pulmonary arterial hypertension (PAH), PH due to left heart disease, PH due to lung disease and/or hypoxia, PH due to chronic blood clots in the lung and PH due to unknown causes (Simonneau et al. 2019).

Mounting evidence indicates the essential role of store-operated calcium entry (SOCE) in the development of PH (Lin et al. 2004; Song et al. 2011; Liu et al. 2012; Smith et al. 2016). SOCE relies on the STIM/Orai/TRPC protein complex. Upon Ca^2+^ store depletion, STIM protein accumulates at the endoplasmic reticulum (ER)-plasma membrane junctions to facilitate the conductance of store-operated calcium channels (SOCs) formed by multimeric Orai subunits (Orai1/2) and/or the functional coupling of Orai and transient receptor potential canonical (TRPC) channels (TRPC1/4/5) (Hogan and Rao 2015). Specifically, increased protein abundance of SOCE and related calcium entry is universally found in pulmonary arterial smooth muscle cells (PASMCs) from diverse PH models and is thought to be responsible for sustained vasoconstriction and vascular remodelling in distal muscular-type pulmonary arteries (Lin et al. 2004; Song et al. 2011; Liu et al. 2012; Smith et al. 2016).

Owing to the progressive development of PH and limitations in diagnostic techniques, elevated PAP and irreversible pulmonary vessel remodelling may exist long before clinical diagnosis (Rich and Rich 2014; Dunlap and Weyer 2016). In view of this, preventive medication neutralising pulmonary vessel disorders would be a necessary and beneficial supplement for PH treatment (Huston et al. 2019). However, the preventive usage of clinical vasodilators (e.g., prostaglandins, phosphodiesterase-5 inhibitors, and endothelin-1 receptor antagonists) is limited by hypotension and other side effects resulting from non-selective vasodilation on the systemic vascular bed (Siobal 2007). Given that preventive medication will be used in PH susceptible individuals or pre-PH patients for a long time, those severe side effects are definitively unacceptable. Therefore, the development of safe and effective PH preventive agents is urgently needed.

As a highly regarded traditional herbal medicine, ginseng and its extracts, have been widely used in the prevention and treatment of various cardiovascular diseases in Asian countries for centuries (Kim 2012; Lee and Kim 2014). Interestingly, several manuscripts have implied a role for ginsenoside Rb1, the main active ingredient of ginseng, in the regulation of intracellular calcium homeostasis and vascular tone (Jiang et al. 2007; Wang et al. 2015). *Ex vivo* experiments from our lab further illustrated that ginsenoside Rb1 depressed pulmonary vasoreactivity by inhibiting SOCE in PASMCs (Wang et al. 2015). Inspired by the literature and preliminary work, we hypothesised that ginsenoside Rb1 may have a preventive effect on PH.

This study explores the possibility of ginsenoside Rb1 as a preventive medication and potential mechanisms involving SOCE using a monocrotaline (MCT)-induced PH rat model. As a classical mimic of type I PH, i.e., PAH, PH gradually develops 3 weeks after MCT injection. To imitate preventive medication, ginsenoside Rb1 was coadministered with MCT for 21 consecutive days. Thereafter, comprehensive parameters related to SOCE, vascular tone, histological changes, hemodynamic parameters, etc., were measured to identify the *in vivo* effects and potential mechanisms of preventive ginsenoside Rb1 medication on PAH.

## Materials and methods

### Monocrotaline induced PAH rat model and ginsenoside Rb1 treatment

To exclude the influences of sex and estrogen, male Sprague-Dawley rats (170-180 g) were used in these experiments (Tofovic 2010; Mair et al. 2014; Batton et al. 2018). The animals obtained from the animal centre of Fujian Medical University (Fuzhou, China) were randomly allocated to Control, MCT and MCT + Rb1 groups (*n* = 20/group). Control rats received only saline injection. Rats in the MCT + Rb1 and MCT groups were intraperitoneally administered single doses of 50 mg/kg MCT combined with 30 mg/kg/day ginsenoside Rb1 or equivalent volumes of saline for 21 consecutive days. The dosages of MCT and ginsenoside Rb1 were determined according to the literature and our preliminary experiments (Jiang et al. 2007; Chan et al. 2011; Lee et al. 2015; Zhang et al. 2015). The animal care and experimental procedures were approved by the Animal Care and Use Committee of Fujian Medical University.

### Hemodynamic measurements and lung histological examination

Rats were anaesthetised using 1 g/kg urethane. Systemic arterial pressure (SAP) was measured using a custom catheter inserted into the left carotid artery. Right ventricle systolic pressure (RVSP) and PAP were measured by advancing a Mikro-Tip pressure catheter (Millar Instruments, TX) into the main pulmonary artery (PA) through direct puncture of the right ventricle, using an open-chest approach as previously described (Mu et al. 2018).

After the hemodynamic measurement, the animals were sacrificed with an overdose of urethane. Heart and lung specimens were immediately collected. The right ventricular mass index (RVMI) was determined by calculating the ratio of the wet weight of the right ventricle to the left ventricular wall plus septum [RV/(LV + S)]. The left lungs were fixed with formaldehyde injected through the trachea, and then embedded in paraffin. Lung cross-sections were subjected to haematoxylin and eosin (H&E) staining. The images of 15 vessels with 51–100 μm o.d. or 101–150 μm o.d. in each group were taken to measure the ratio of wall thickness to the radius and lumen area to the total area using Image-Pro 6.0 software.

### Isometric contraction of the PA ring

The third- and fourth-generation PAs (300–800 µm o.d.) were carefully dissociated from the lungs and cut into 4 mm length rings. After endothelium disruption, the arterial rings were placed into modified Krebs solution (118 mM NaCl, 4.7 mM KCl, 1.2 mM MgCl_2_, 10 mM HEPES, 10 mM glucose, 2 mM CaCl_2_, pH 7.2) saturated by 95% O_2_ plus 5% CO_2_ at 37 °C. The isometric contraction was measured by suspending PA rings between two stainless steel stirrups with a resting tension of 1.0 g. Data were collected by a force transducer connected to an RM6240 polygraph (Chengyi, Chengdu, China). Next, 60 mM KCl and 3 × 10^−7^ M phenylephrine followed by 10^−5^ M acetylcholine were added into the bath solution to verify the maximum contraction of PA rings and complete disruption of the endothelium. Those with >20% acetylcholine-induced relaxations were excluded from the experiment.

### Pulmonary arterial smooth muscle cell culture

Primary cultures of PASMCs were enzymatically isolated from third- and fourth-generation PAs as previously described (Liu et al. 2012; Wang et al. 2015; Mu et al. 2018). Deendothelialized arteries were placed into HEPES-buffered salt solution (HBSS) (130 mM NaCl, 5 mM KCl, 1.2 mM MgCl_2_, 20 µM CaCl_2,_ 10 mM HEPES, 10 mM glucose, pH 7.4) containing 1750 U/mL type I collagenase, 9.5 U/mL papain, 2 mg/mL bovine serum albumin and 1 mM dithiothreitol at 37 °C for 20 min. PASMCs were gently dispersed in Ca^2+^-free HBSS at room temperature. Cells attached to glass coverslips were transiently cultured in Ham’s F-12 medium supplemented with 0.5% fetal calf serum.

### Measurement of [Ca^2+^]_i_ in PASMCs

Coverslips with PASMCs were loaded with 10 µM fluo-3 AM in normal Tyrode solution (137 mM NaCl, 5.4 mM KCl, 2 mM CaCl_2_, 1 mM MgCl_2_, 10 mM HEPES, 10 mM glucose, pH 7.4) for 1 h at room temperature. After thorough flushing to remove the extracellular dye and 30 min of rest to complete cytosolic dye de-esterification, fluo-3 AM fluorescence was excited at 488 nm, and emission light at >515 nm was detected. Data were collected online with an analog-to-digital interface (PTI FeliX32, Horiba Scientific, NJ). [Ca^2+^]_i_ was calculated as follows: [Ca^2+^]_i_ = *K*_d_·(*F* − *F*_bg_)/(*F*_max_ − *F*), where *F*_bg_ indicates background fluorescence and F_max_ indicates the maximum fluorescence determined *in situ* in cells superfused with 10 µM 4-bromo-A-23187 and 10 mM Ca^2+^. The *K*_d_ of fluo-3 AM is 1.1 µM.

### Quantitative real-time PCR

Total RNA was extracted from deendothelialized distal PAs using TRIzol (Invitrogen, CA). cDNA was synthesized from total RNA using the Transcriptor First Strand cDNA Synthesis Kit (Roche, Basel, Switzerland). The mRNA expression of STIM, Orai, and TRPCs was determined by the FastStart DNA Master SYBR Green I Kit and LightCycler 2.0 (Roche, Basel, Switzerland). Data were normalized to β-actin as the reference gene. The sequences of the primers were as follows: 5′-GCTTTCCCTGGAGGACTCTT-3′ (sense) and 5′-GGGATGCCACTAGAGAGCTG-3′ (antisense) for STIM2; 5′-ATGGTAGCGATGGTGGAAGT-3′ (sense) and 5′-ACGGAGTTGAGGTTGTGGAC-3′ (antisense) for Orai1; 5′-AATGGGACATACTGCCAAGC-3′ (sense) and 5′-TGCCAAACAAACAAACCAAA-3′ (antisense) for Orai2; 5′-AGCCTCTTGACAAACGAGGA-3′ (sense) and 5′-TGACATCTGTCCGAACCAAA-3′ (antisense) for TRPC1; 5′-GCGTGCTGCTGATAACTTGA-3′ (sense) and 5′-CGAAGCGGAAGCTAGAAATG-3′ (antisense) for TRPC4;5′-CCCATCTATGAGGGTTACGC-3′ (sense) and 5′-TTTAATGTCACGCACGATTTC-3′ (antisense) for β-actin.

### Western blot analysis

The total protein extracted from deendothelialized distal PA homogenate was subjected to 12% SDS-PAGE and then transferred to a polyvinylidene fluoride membrane (Millipore, MA). Following blockade with 5% BSA buffer for 2 h, membranes were probed with anti-STIM2 (1:200; Alomone Labs, Jerusalem, Israel), anti-Orai1 (1:200; Alomone Labs, Jerusalem, Israel), anti-Orai2 (1:200; Alomone Labs, Jerusalem, Israel), anti-TRPC1 (1:300; Abcam, MA), anti-TRPC4 (1:200; Alomone Labs, Jerusalem, Israel), anti-β-actin (1:500; Cell Signalling Technology, MA) primary antibodies overnight and horseradish peroxidase (HRP)-conjugated secondary antibodies (1:5000; Cell Signalling Technology, MA) for 1 h. The bands were visualized by enhanced chemiluminescence reagent (Thermo Fisher Scientific, CA). The optical density of each blot was quantified using Quantity One software (Bio-Rad, CA) and normalized to β-actin on the same membrane.

### Solutions and reagents

Ginsenoside Rb1 (purity ≥ 98%) was purchased from Tianzhilan Biotechnology Co., Ltd. (Jining, China). All other reagents were purchased from Sigma Chemical Co. (MO). Ginsenoside Rb1 was dissolved in saline. Nifedipine (Nif), cyclopiazonic acid (CPA) and fluo-3 AM were dissolved in dimethyl sulfoxide (DMSO). MCT was dissolved in 1 M HCl, neutralized to pH 7.4. All other reagents were dissolved in sterile water.

### Statistical analysis

Data were presented as the mean ± standard deviation (SD), and “*n*” indicates the number of samples used. Curve graphing was performed using SigmaPlot 11.0 software. Statistical comparison was performed by unpaired or paired Student’s *t*-tests and ANOVA as appropriate (SPSS, version 21.0). A value of *p* < 0.05 was considered statistically significant.

## Results

### Preventive treatment with ginsenoside Rb1 reduced the characteristic changes of MCT-induced PAH in rats

**Figure 1.**
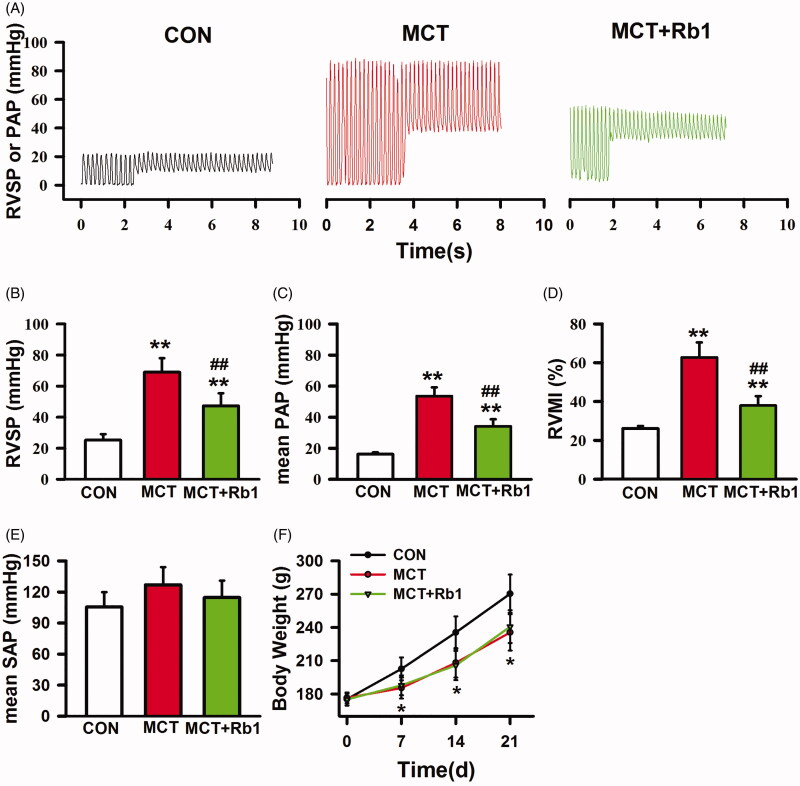
Effects of ginsenoside Rb1 preventive medication on hemodynamic parameters and body weight of MCT-induced PH rats. (A) shows the representative traces of RVSP and PAP. (B), (C), (D) and (E) are bar graphs showing RVSP, mean PAP, RVMI, and mean SAP in each group. (F) shows the body weights of rats measured weekly. Data are presented as mean ± SD (*n* = 20 each). **p < 0.01 vs. Control; ##*p* < 0.01 vs. MCT.

We first assessed the effect of preventive treatment with ginsenoside Rb1 on hemodynamic parameters. As shown in Figure 1, PH developed 3 weeks after MCT injection, as indicated by elevated RVSP (68.9 ± 9.0 vs. 25.2 ± 3.9 mmHg, MCT vs. Control, *p* < 0.01, Figure 1(A,B)), increased mean PAP (53.6 ± 5.6 mmHg vs. 16.1 ± 1.1 mmHg, MCT vs. Control, *p* < 0.01, Figure 1(A,C)) and RVMI (0.628 ± 0.077 vs. 0.261 ± 0.013, MCT vs. Control, *p* < 0.01, Figure 1(D)). As a result of ginsenoside Rb1 preventive medication, the values of the RVSP (47.3 ± 8.1 mmHg, MCT + Rb1 vs. MCT, *p* < 0.01, Figure 1(A,B)), mean PAP (34.1 ± 4.5 mmHg, MCT + Rb1 vs. MCT, *p* < 0.01, Figure 1(A,C)) and RVMI (0.380 ± 0.047, MCT + Rb1 vs. MCT, *p* < 0.01, Figure 1(D)) were significantly reduced compared with administration of MCT alone.

**Figure 2.**
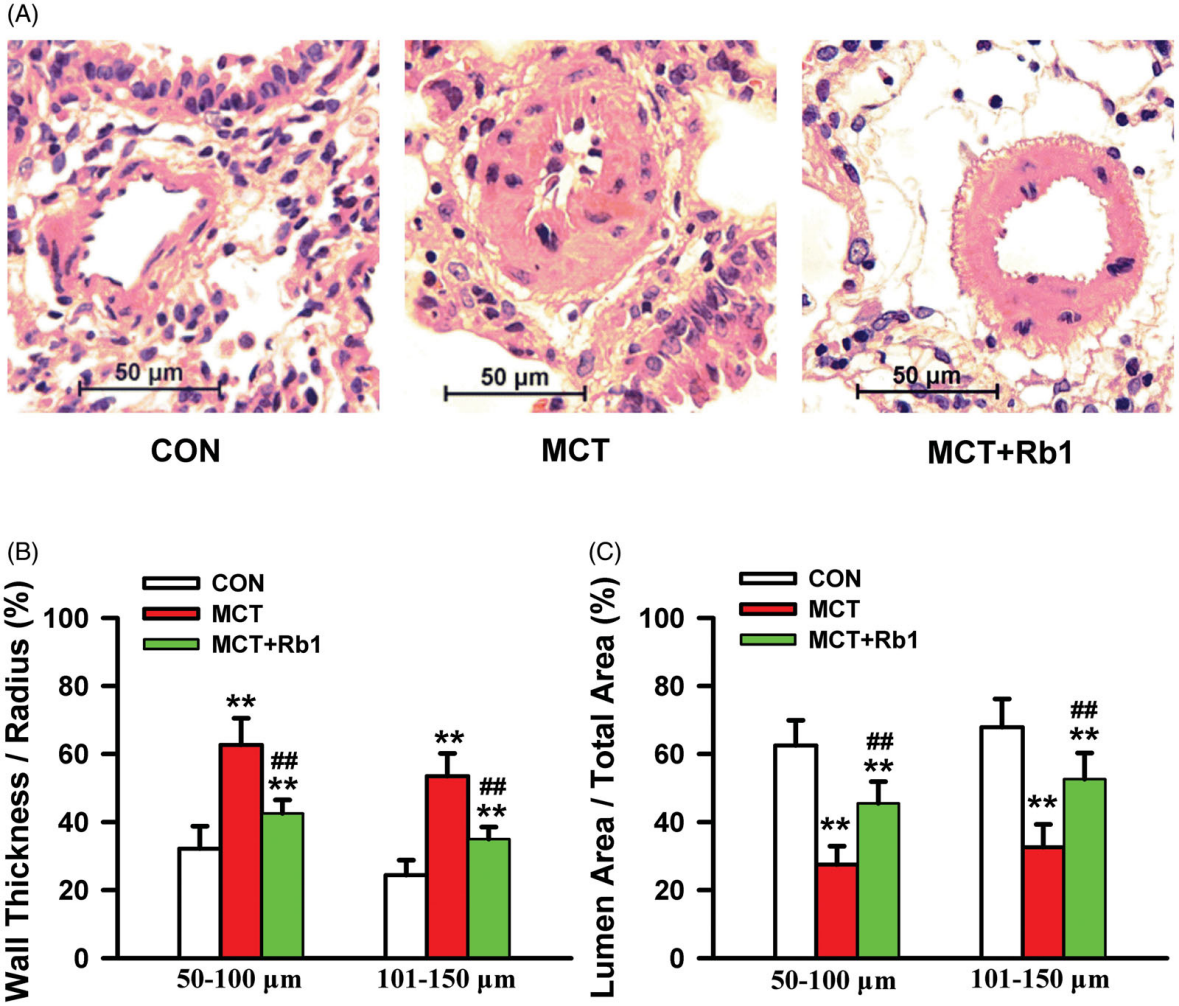
Effects of ginsenoside Rb1 preventive medication on MCT-induced pulmonary vascular remodelling. (A) shows the representative images of H&E staining on lung cross-sections of each group rats. Magnification × 40. (B) and (C) are bar graphs showing wall thickness/radius ratio and luminal area/total area ratio in each group. Data are presented as mean ± SD (*n* = 15 each). **p < 0.01 vs. Control; ##*p* < 0.01 vs. MCT.

Histological examination of lung sections from MCT-treated rats showed that the distal pulmonary arterial walls, especially the tunica media, were dramatically thickened compared with those of saline control animals (Figure 2(A)). Morphometric analysis of these vessels showed that MCT increased the wall thickness/radius ratio (50–100 μm: 0.627 ± 0.078 *vs.* 0.322 ± 0.066; 101–150 μm: 0.535 ± 0.067 *vs.* 0.244 ± 0.044. MCT vs. Control, *p* < 0.01, Figure 2(B)), while diminishing the luminal area/total area ratio (50–100 μm: 0.275 ± 0.054 *vs.* 0.625 ± 0.074; 101–150 μm: 0.326 ± 0.067 vs. 0.679 ± 0.083. MCT vs. Control, *p* < 0.01, Figure 2(C)). Ginsenoside Rb1 relieved pulmonary arterial wall thickening (Figure 2(A)), reduced the thickness/radius ratio (50–100 μm: 0.425 ± 0.040; 101–150 μm: 0.350 ± 0.036. MCT + Rb1 vs. MCT, *p* < 0.01, Figure 2(B)) and increased the ratio of luminal area/total area (50–100 μm: 0.455 ± 0.064; 101–150 μm: 0.526 ± 0.077. MCT + Rb1 vs. MCT, *p* < 0.01, Figure 2(C)) in MCT-treated rats. These results indicate that preventive treatment with ginsenoside Rb1 significantly reduces pulmonary vascular remodelling in MCT-induced PAH rats.

Despite significant amelioration of pulmonary hemodynamic status and right ventricle hypertrophy, our results also hinted that ginsenoside Rb1 had no effect on either SAP or body weight gain in rats treated with MCT. Inconsistent with the decreased PAP, ginsenoside Rb1 did not decrease systemic arterial pressure, as evidenced by very similar mean SAP values in the MCT and MCT + Rb1 groups (mean SAP: 114.6 ± 16.3 vs. 126.8 ± 17.2 mmHg, MCT + Rb1 vs. MCT, *p* > 0.05, Figure 1(E)). The body weights of rats were measured 7, 14, and 21 days after saline or MCT injection. As shown in Figure 1(F), steady weight gain was severely curtailed by MCT (MCT vs. Control, *p* < 0.05), whereas no significant difference was found between the MCT + Rb1 and MCT groups (MCT + Rb1 vs. MCT, *p* > 0.05).

### Preventive treatment with ginsenoside Rb1 inhibited SOCE complex expression in PAs of MCT-induced PAH rats

Because the *in vivo* effect of ginsenoside Rb1 on PAH had been addressed, we further investigated its effect on SOCE with the expectation of providing an explanation for its pharmacological efficacies.

**Figure 3.**
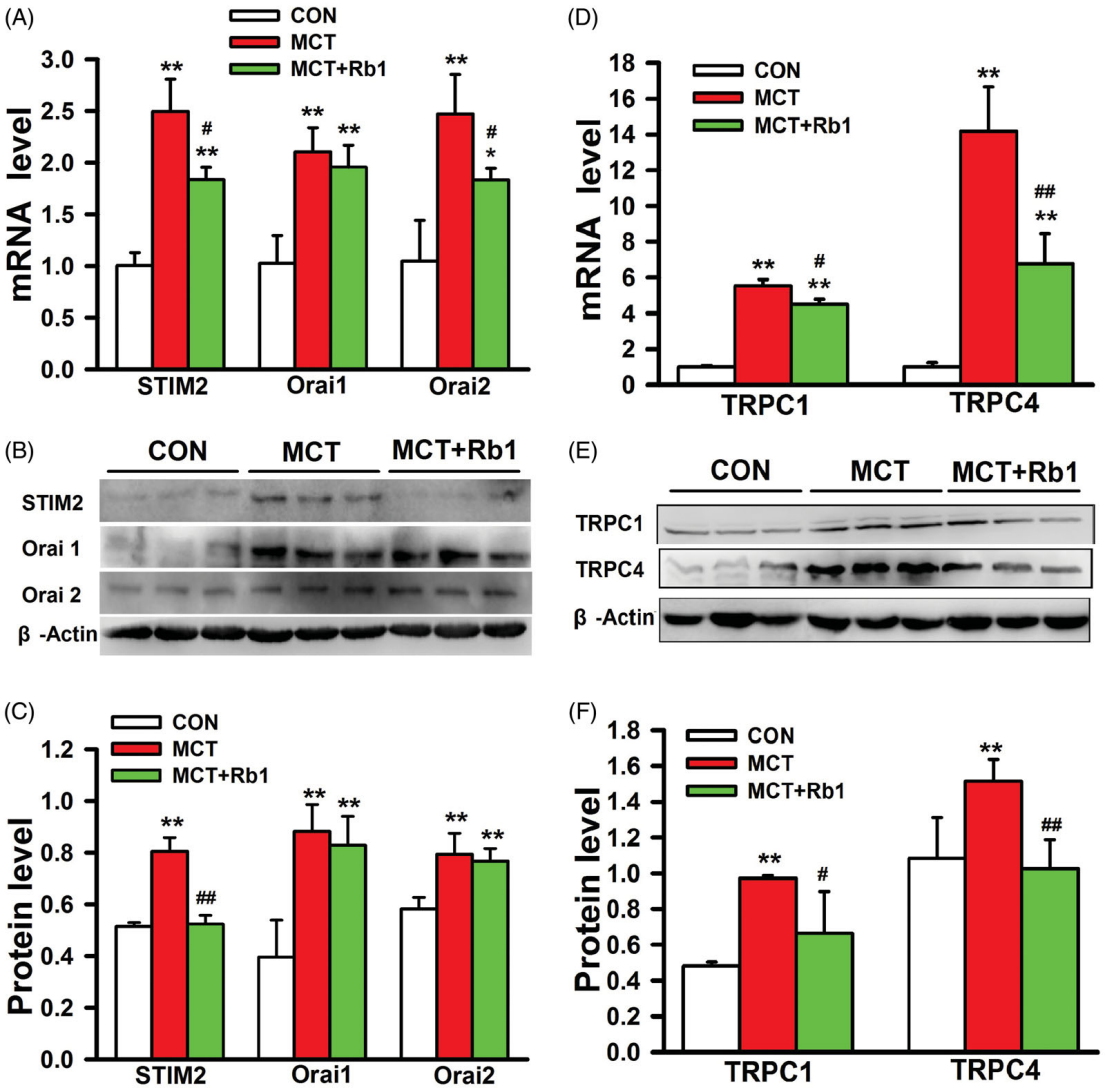
Effects of ginsenoside Rb1 on SOCE complex expression in PAs derived from MCT-induced PH rats. (A), (B) and (C) show mRNA relative expression, representative western blotting and relative protein intensity of STIM2, Orai1 and Orai2. (D), (E) and (F) show mRNA relative expression, representative western blotting and relative protein intensity of TRPC1, TRPC4. Data are presented as mean ± SD (*n* = 5 each for qPCR, *n* = 6 each for Western blotting). *p < 0.05 and **p < 0.01 vs. Control; #*p* < 0.05, ##*p* < 0.01, vs. MCT.

Consistent with previous reports, we found that the expression of SOCE complex components was substantially increased in MCT-treated rats (Song et al. 2011; Liu et al. 2012; Rode et al. 2018). The mRNA and protein content of STIM2 (mRNA: 2.50 ± 0.31 vs. 1.01 ± 0.12; protein: 0.80 ± 0.05 vs. 0.51 ± 0.01. MCT vs. Control, *p* < 0.01, Figure 3(A–C)), Orai1 (mRNA: 2.10 ± 0.23 vs. 1.03 ± 0.27; protein: 0.88 ± 0.10 vs. 0.40 ± 0.14. MCT vs. Control, *p* < 0.01, Figure 3(A–C)), Orai2 (mRNA: 2.47 ± 0.38 vs. 1.05 ± 0.39; protein: 0.79 ± 0.08 vs. 0.58 ± 0.04. MCT *vs.* Control, *p* < 0.01, Figure 3(A–C)), TRPC1 (mRNA: 5.52 ± 0.37 vs. 1.00 ± 0.08; protein: 0.97 ± 0.02 *vs*. 0.48 ± 0.02. MCT vs. Control, *p* < 0.01, Figure 3(D–F)) and TRPC4 (mRNA: 14.28 ± 2.48 vs. 1.02 ± 0.22; protein: 1.52 ± 0.12 vs. 1.08 ± 0.23. MCT vs. Control, *p* < 0.01, Figure 3(D–F)) were significantly greater in distal PAs derived from MCT-treated rats.

Ginsenoside Rb1 markedly suppressed MCT-induced mRNA expression of STIM2 (1.84 ± 0.12, MCT + Rb1 vs. MCT, *p* < 0.05, Figure 3(A)), Orai2 (1.83 ± 0.11, MCT + Rb1 *vs.* MCT, *p* < 0.05, Figure 3(A)), TRPC1 (4.51 ± 0.28, MCT + Rb1 *vs.* MCT, *p* < 0.05, Figure 3(D)) and TRPC4 (6.76 ± 1.70, MCT + Rb1 vs. MCT, *p* < 0.01, Figure 3(D)), and protein expression of STIM2 (0.52 ± 0.03, MCT + Rb1 vs. MCT, *p* < 0.05), TRPC1 (0.66 ± 0.23, MCT + Rb1 vs. MCT, *p* < 0.05, Figure 3(E,F)) and TRPC4 (1.03 ± 0.16, MCT + Rb1 vs. MCT, *p* < 0.01, Figure 3(E,F)) in distal PAs.

### Preventive treatment with ginsenoside Rb1 attenuated SOCE-mediated Ca^2+^ transients in PASMCs derived from MCT-induced PAH rats

**Figure 4.**
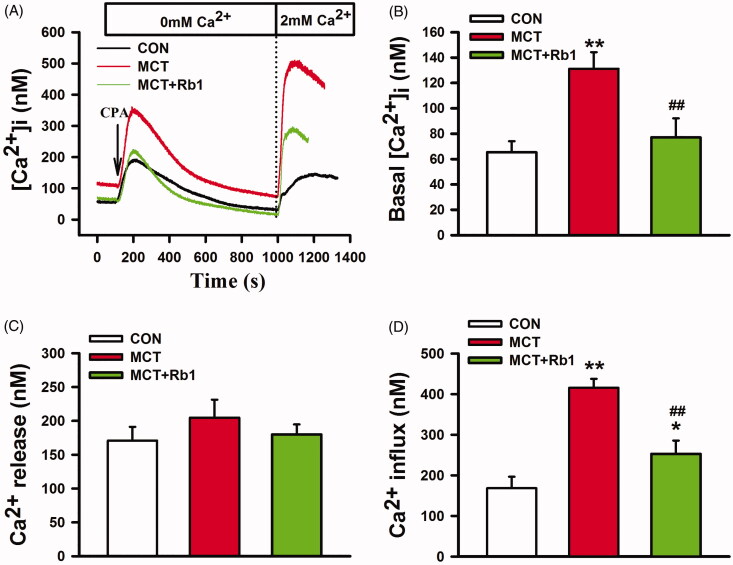
Effects of ginsenoside Rb1 on SOCE related Ca^2+^ transients in PASMCs derived from MCT-induced PH rats. (A) shows the representative traces of CPA-induced Ca^2+^ transients. (B), (C) and(D) are bar graphs showing basal [Ca^2+^]i, peak changes of CPA-induced Ca^2+^ release and Ca^2+^ entry, respectively. Data are presented as mean ± SD (*n* = 15 each). *p < 0.05 and **p < 0.01 vs. Control; ##*p* < 0.01 vs. MCT.

CPA was introduced here to deplete ER Ca^2+^ stores and activate SOCE. PASMCs were treated with 10 µM CPA for 10 min in Ca^2+^-free Tyrode solution (containing 0.1 mM EGTA and 3 µM Nif) followed by replenishment of 2 mM Ca^2+^ to the external solution. The basal [Ca^2+^]_i_ and the amplitude of the CPA induced Ca^2+^ influx were significantly increased in the MCT group (basal [Ca^2+^]_i_: 131.3 ± 13.0 nM vs. 65.4 ± 8.7 nM; Ca^2+^ influx: 415.6 ± 22.2 nM vs. 168.5 ± 28.2 nM. MCT vs. Control, *p* < 0.01, Figure 4(B,D)), whereas the CPA induced Ca^2+^ release was similar to its normal control (Figure 4(C)). Ginsenoside Rb1 significantly depressed MCT-induced elevation in basal [Ca^2+^]_i_ and Ca^2+^ influx (basal [Ca^2+^]_i_: 77.1 ± 15.0 nM; Ca^2+^ influx: 253.0 ± 32.6 nM. MCT + Rb1 vs. MCT, *p* < 0.01, Figure 4(B,D)).

### Preventive treatment with ginsenoside Rb1 attenuated SOCE-mediated PA contraction in MCT-induced PAH rats

**Figure 5.**
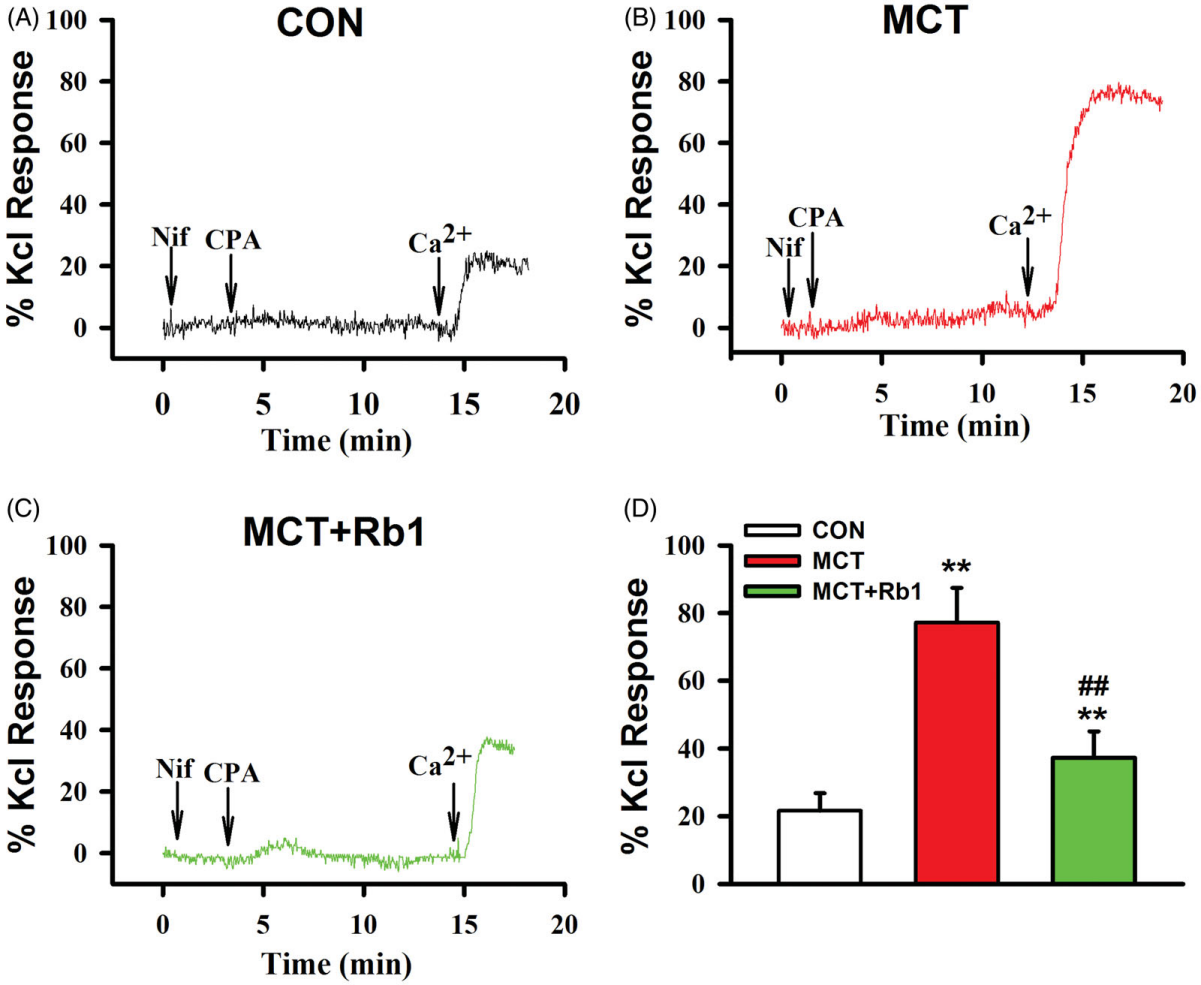
Effects of ginsenoside Rb1 on SOCE mediated PAs contraction in MCT-induced PH rats. (A), (B) and (C) show the representative traces of CPA-induced PA contraction. (D) is the average percentage values of CPA induced maximum contraction over 60 mM KCl induced contraction. Data are presented as mean ± SD (*n* = 14 in Control, *n* = 13 in MCT and MCT + Rb1 each). **p < 0.01 vs. Control; ##*p* < 0.01 vs. MCT.

PA rings were placed in Ca^2+^-free modified Krebs solution containing 3 µM Nif. After exposure to 10 µM CPA for 10 min, SOCE mediated contraction was initiated by the readmission of 2 mM Ca^2+^. The CPA induced maximum contraction response was significantly enhanced in PAs from MCT-treated rats (0.772 ± 0.102 vs. 0.216 ± 0.051. MCT vs. Control, *p* < 0.01, Figure 5(A–D)). Preventive treatment with ginsenoside Rb1 significantly decreased CPA-induced PA contraction (0.373 ± 0.078, MCT + Rb1 vs. MCT, *p* < 0.01, Figure 5(C,D)).

## Discussion

This study assessed the effect of preventive medication with ginsenoside Rb1 on MCT-induced PAH and its potential mechanisms. We demonstrated that ginsenoside Rb1 reduced SOCE complex expression and related calcium entry, depressed PA contraction, which may yield significant amelioration in pulmonary hemodynamic parameters, pulmonary arterial wall thickening, and right ventricle hypertrophy in MCT-induced PAH rats.

In this study, MCT treated rats successfully recapitulated a similar expression pattern of SOCE complex components previously reported by our lab (Liu et al. 2012). Namely, STIM2, Orai1, Orai2, TRPC1, and TRPC4 contents were significantly elevated in deendothelialized distal PA samples. The protein contents of STIM2, TRPC1, and TRPC4 returned to their normal levels in the presence of ginsenoside Rb1. Accompanying the modified SOCE complex expression, ginsenoside Rb1 reduced CPA-induced calcium influx by 39.12%, further yielding a clean rescue of soared basal [Ca^2+^]_i_ (showing no significant difference from the normal control). Combined with the results we previously reported, the present study further confirmed the antagonism of ginsenoside Rb1 on SOCE (Wang et al. 2015).

Enhanced SOCE in PASMCs is recognized as the primary contributor to PH characteristic pulmonary vascular constriction and remodelling (Wang et al. 2009; Wang et al. 2015; Jiao et al. 2016; Mu et al. 2018; Song et al. 2018). In this study, MCT caused excessive contraction of PAs, whereas the increase in vascular tone was reduced by 71.72% in the presence of ginsenoside Rb1. In addition, thickening of the distal pulmonary arterial walls, a typical histological change of PH, was also reduced, as evidenced by decreased wall thickness/radius ratio and increased lumen area/total area ratio. Given that inhibition of SOCE by ginsenoside Rb1 has been confirmed by *ex vivo* and *in vivo* experiments, it is logically reasonable that ginsenoside Rb1 mediated restriction on pulmonary vascular tone and remodelling is at least partially attributed to SOCE inhibition.

Fatal right heart failure, which is secondary to increased right ventricular afterload and irreversible ventricular remodelling, is the worst outcome of PH (Thenappan et al. 2016). Ginsenoside Rb1 antagonized the increases in PAP and RVSP by 36.38 and 31.40%, respectively. Meanwhile, the RVMI decreased by 39.50%. Through its effective counteraction of increased PAP and right ventricular remodelling, ginsenoside Rb1 may contribute to clinical prevention or amelioration of PH-induced right heart failure. The current treatment for PH is a combination of specific and primary therapy. Essentially, PH-specific therapy sets to decrease pulmonary circulation pressure by directly inducing pulmonary vasodilation, whereas primary therapy targets the causes and complications of PH (Fuso et al. 2011; Cottin et al. 2014). Since SOCE and subsequent pulmonary vasoconstriction, the target of ginsenoside Rb1, have been recognized as a common mechanism of PH, we speculated that ginsenoside Rb1 would be PH-specific and generally effective for various forms of PH (Song et al. 2011; Liu et al. 2012; Smith et al. 2016).

Stubborn hypotension is a common side effect of vasodilator treatment, resulting from non-selective vasodilation (Siobal 2007). Interestingly, preventive treatment with ginsenoside Rb1 did not affect the mean SAP of PAH rats. This result probably hinted at selective vasodilation of the pulmonary vascular bed, which deserves further attention. Despite markedly mitigating hemodynamic and histological perturbations of PAH, ginsenoside Rb1 contributed little to body weight gain in this study. The precise reasons for MCT-induced body mass change remain unclear. There is a view that body mass change might be a byproduct of systemic toxicity of MCT; i.e., that MCT causes not only PAH but also damage to many other organs (Roth et al. 1981; Copple et al. 2004; Yao et al. 2014; Kim et al. 2018; Lee et al. 2019). Our result may imply less involvement of PAH in MCT-induced body mass change.

It should be noted that this study is a preliminary demonstration that ginsenoside Rb1 may help prevent PH. The pharmacological preventive efficiency of ginsenoside Rb1 has not yet been fully described without further information about the effects on PH pathological progression and the dose-effect relationship. Similarly, we merely illustrated a possible mechanism for ginsenoside Rb1 in preventing PH via SOCE inhibition, which awaits further confirmation by direct evidence targeting the STIM/Orai/TRPC pathway. In addition, the possible toxicological effects of ginsenoside Rb1 as a long-term preventive medication cannot be excluded until it is thoroughly studied in rats.

The limitations of the experimental model should also be taken into account. The conventional MCT-induced PAH model has a short survival period, usually less than one month (Lachant et al. 2018). Its subacute process cannot perfectly simulate the progressive development of human PH (Zhuang et al. 2018). In addition, MCT-induced PAH appears to be preventable or curable by several agents, which is quite different from the case of human PH (Ruiter et al. 2013; Colvin and Yeager 2014).

## Conclusion

The present study provided evidence for the application of ginsenoside Rb1 to prevent MCT-induced PAH by inhibiting SOCE and related pulmonary vasoconstriction. Although follow-up studies on different time points, precise mechanisms, and better administration modes using multiple PH models should be completed before drawing a conclusion, ginsenoside Rb1 is a promising candidate for PH-specific prevention that deserves further investigation.

## References

[CIT0001] Batton KA, Austin CO, Bruno KA, Burger CD, Shapiro BP, Fairweather D. 2018. Sex differences in pulmonary arterial hypertension: role of infection and autoimmunity in the pathogenesis of disease. Biol Sex Differ. 9(1):15. 29669571 10.1186/s13293-018-0176-8 PMC5907450

[CIT0002] Chan PC, Peckham JC, Malarkey DE, Kissling GE, Travlos GS, Fu PP. 2011. Two-year toxicity and carcinogenicity studies of *Panax ginseng* in Fischer 344 rats and B6C3F1 mice. Am J Chin Med. 39(4):779–788. 21721156 10.1142/S0192415X11009184 PMC10214765

[CIT0003] Colvin KL, Yeager ME. 2014. Animal models of pulmonary hypertension: matching disease mechanisms to etiology of the human disease. J Pulm Respir Med. 4(4):198. 25705569 10.4172/2161-105X.1000198 PMC4334132

[CIT0004] Copple BL, Rondelli CM, Maddox JF, Hoglen NC, Ganey PE, Roth RA. 2004. Modes of cell death in rat liver after monocrotaline exposure. Toxicol Sci. 77(1):172–182. 14600277 10.1093/toxsci/kfh011

[CIT0005] Cottin V, Lorillou M, Khouatra C, Traclet J, Nesme P, Cordier JF. 2014. Pulmonary hypertension-specific therapy in patients with chronic respiratory insufficiency. Eur Respir J. 44(3):819–821. 25176957 10.1183/09031936.00054214

[CIT0006] Dunlap B, Weyer G. 2016. Pulmonary hypertension: diagnosis and treatment. Am Fam Physician. 94(6):463–469. 27637122

[CIT0007] Fuso L, Baldi F, Di Perna A. 2011. Therapeutic strategies in pulmonary hypertension. Front Pharmacol. 2:21. 21687513 10.3389/fphar.2011.00021 PMC3108478

[CIT0008] Hogan PG, Rao A. 2015. Store-operated calcium entry: Mechanisms and modulation. Biochem Biophys Res Commun. 460(1):40–49. 25998732 10.1016/j.bbrc.2015.02.110 PMC4441756

[CIT0009] Huetsch JC, Suresh K, Bernier M, Shimoda LA. 2016. Update on novel targets and potential treatment avenues in pulmonary hypertension. Am J Physiol Lung Cell Mol Physiol. 311(5):L811–L831. 27591245 10.1152/ajplung.00302.2016 PMC5130539

[CIT0010] Huston JH, Frantz RP, Brittain EL. 2019. Early intervention: Should we conduct therapeutic trials for mild pulmonary hypertension before onset of symptoms?. Pulm Circ. 9(2):2045894019845615. 10.1177/2045894019844994 PMC6469279 30931829

[CIT0011] Jiang QS, Huang XN, Dai ZK, Yang GZ, Zhou QX, Shi JS, Wu Q. 2007. Inhibitory effect of ginsenoside Rb1 on cardiac hypertrophy induced by monocrotaline in rat. J Ethnopharmacol. 111(3):567–572. 17374466 10.1016/j.jep.2007.01.006

[CIT0012] Jiao HX, Mu YP, Gui LX, Yan FR, Lin DC, Sham JS, Lin MJ. 2016. Increase in caveolae and caveolin-1 expression modulates agonist-induced contraction and store- and receptor-operated Ca^(2+)^ entry in pulmonary arteries of pulmonary hypertensive rats. Vascul Pharmacol. 84:55–66. 27311393 10.1016/j.vph.2016.06.004

[CIT0013] Kim JH. 2012. Cardiovascular diseases and *Panax ginseng*: a review on molecular mechanisms and medical applications. J Ginseng Res. 36(1):16–26. 23717100 10.5142/jgr.2012.36.1.16 PMC3659571

[CIT0014] Kim KH, Kim HK, Chan SY, Kim YJ, Sohn DW. 2018. Hemodynamic and histopathologic benefits of early treatment with macitentan in a rat model of pulmonary arterial hypertension. Korean Circ J. 48(9):839–853. 30088353 10.4070/kcj.2017.0394 PMC6110709

[CIT0015] Lachant DJ, Meoli DF, Haight D, Lyons JA, Swarthout RF, White RJ. 2018. Low dose monocrotaline causes a selective pulmonary vascular lesion in male and female pneumonectomized rats. Exp Lung Res. 44(1):51–61. 29381088 10.1080/01902148.2017.1422157 PMC6240164

[CIT0016] Lee CH, Kim JH. 2014. A review on the medicinal potentials of ginseng and ginsenosides on cardiovascular diseases. J Ginseng Res. 38(3):161–166. 25378989 10.1016/j.jgr.2014.03.001 PMC4213864

[CIT0017] Lee H, Yeom A, Kim KC, Hong YM. 2019. Effect of ambrisentan therapy on the expression of endothelin receptor, endothelial nitric oxide synthase and NADPH oxidase 4 in monocrotaline-induced pulmonary arterial hypertension rat model. Korean Circ J. 49(9):866–876. 31165592 10.4070/kcj.2019.0006 PMC6713827

[CIT0018] Lee SM, Bae BS, Park HW, Ahn NG, Cho BG, Cho YL, Kwak YS. 2015. Characterization of Korean red ginseng (*Panax ginseng* Meyer): history, preparation method, and chemical composition. J Ginseng Res. 39(4):384–391. 26869832 10.1016/j.jgr.2015.04.009 PMC4593794

[CIT0019] Lin MJ, Leung GP, Zhang WM, Yang XR, Yip KP, Tse CM, Sham JS. 2004. Chronic hypoxia-induced upregulation of store-operated and receptor-operated Ca^2+^ channels in pulmonary arterial smooth muscle cells: a novel mechanism of hypoxic pulmonary hypertension. Circ Res. 95(5):496–505. 15256480 10.1161/01.RES.0000138952.16382.ad

[CIT0020] Liu XR, Zhang MF, Yang N, Liu Q, Wang RX, Cao YN, Yang XR, Sham JS, Lin MJ. 2012. Enhanced store-operated Ca^2+^ entry and TRPC channel expression in pulmonary arteries of monocrotaline-induced pulmonary hypertensive rats. Am J Physiol, Cell Physiol. 302(1):C77–C87. 21940663 10.1152/ajpcell.00247.2011 PMC3328901

[CIT0021] Mair KM, Wright AF, Duggan N, Rowlands DJ, Hussey MJ, Roberts S, Fullerton J, Nilsen M, Loughlin L, Thomas M, et al. 2014. Sex-dependent influence of endogenous estrogen in pulmonary hypertension. Am J Respir Crit Care Med. 190(4):456–467. 24956156 10.1164/rccm.201403-0483OC PMC4214128

[CIT0022] Mu YP, Lin DC, Zheng SY, Jiao HX, Sham JSK, Lin MJ. 2018. Transient receptor potential melastatin-8 activation induces relaxation of pulmonary artery by inhibition of store-operated calcium entry in normoxic and chronic hypoxic pulmonary hypertensive rats. J Pharmacol Exp Ther. 365(3):544–555. 29622593 10.1124/jpet.117.247320

[CIT0023] Rich JD, Rich S. 2014. Clinical diagnosis of pulmonary hypertension. Circulation. 130(20):1820–1830. 25385937 10.1161/CIRCULATIONAHA.114.006971

[CIT0024] Rode B, Bailey MA, Marthan R, Beech DJ, Guibert C. 2018. Orai channels as potential therapeutic targets in pulmonary hypertension. Physiology (Bethesda). 33(4):261–268. 29897302 10.1152/physiol.00016.2018

[CIT0025] Roth RA, Dotzlaf LA, Baranyi B, Kuo CH, Hook JB. 1981. Effect of monocrotaline ingestion on liver, kidney, and lung of rats. Toxicol Appl Pharmacol. 60(2):193–203. 6792747 10.1016/0041-008x(91)90223-2

[CIT0026] Ruiter G, de Man FS, Schalij I, Sairras S, Grunberg K, Westerhof N, van der Laarse WJ, Vonk-Noordegraaf A. 2013. Reversibility of the monocrotaline pulmonary hypertension rat model. Eur Respir J. 42(2):553–556. 23904554 10.1183/09031936.00012313

[CIT0027] Simonneau G, Montani D, Celermajer DS, Denton CP, Gatzoulis MA, Krowka M, Williams PG, Souza R. 2019. Haemodynamic definitions and updated clinical classification of pulmonary hypertension. Eur Respir J. 53(1):1801913. 30545968 10.1183/13993003.01913-2018 PMC6351336

[CIT0028] Siobal MS. 2007. Pulmonary vasodilators. Respir Care. 52(7):885–899. 17594732

[CIT0029] Smith KA, Ayon RJ, Tang H, Makino A, Yuan JX. 2016. Calcium-sensing receptor regulates cytosolic [Ca^2+^] and plays a major role in the development of pulmonary hypertension. Front Physiol. 7:517. 27867361 10.3389/fphys.2016.00517 PMC5095111

[CIT0030] Song MY, Makino A, Yuan JX. 2011. Stim2 contributes to enhanced store-operated Ca entry in pulmonary artery smooth muscle cells from patients with idiopathic pulmonary arterial hypertension. Pulm Circ. 1(1):84–94. 21709766 10.4103/2045-8932.78106 PMC3121304

[CIT0031] Song S, Carr SG, McDermott KM, Rodriguez M, Babicheva A, Balistrieri A, Ayon RJ, Wang J, Makino A, Yuan JX. 2018. Stim2 (stromal interaction molecule 2)-mediated increase in resting cytosolic free Ca^2+^ concentration stimulates PASMC proliferation in pulmonary arterial Hypertension. Hypertension. 71(3):518–529. 29358461 10.1161/HYPERTENSIONAHA.117.10503 PMC5955710

[CIT0032] Thenappan T, Prins KW, Pritzker MR, Scandurra J, Volmers K, Weir EK. 2016. The critical role of pulmonary arterial compliance in pulmonary hypertension. Ann Am Thorac Soc. 13(2):276–284. 26848601 10.1513/AnnalsATS.201509-599FR PMC5461956

[CIT0033] Tofovic SP. 2010. Estrogens and development of pulmonary hypertension: Interaction of estradiol metabolism and pulmonary vascular disease. J Cardiovasc Pharmacol. 56(6):696–708. 20881610 10.1097/FJC.0b013e3181f9ea8d PMC3027839

[CIT0034] Wang C, Li JF, Zhao L, Liu J, Wan J, Wang YX, Wang J, Wang C. 2009. Inhibition of soc/Ca^2+^/nfat pathway is involved in the anti-proliferative effect of sildenafil on pulmonary artery smooth muscle cells. Respir Res. 10:123. 20003325 10.1186/1465-9921-10-123 PMC2797778

[CIT0035] Wang RX, He RL, Jiao HX, Dai M, Mu YP, Hu Y, Wu ZJ, Sham JS, Lin MJ. 2015. Ginsenoside Rb1 attenuates agonist-induced contractile response via inhibition of store-operated calcium entry in pulmonary arteries of normal and pulmonary hypertensive rats. Cell Physiol Biochem. 35(4):1467–1481. 25791507 10.1159/000373966

[CIT0036] Yao J, Li CG, Gong LK, Feng CC, Li CZ, Gao M, Luan Y, Qi XM, Ren J. 2014. Hepatic cytochrome p450s play a major role in monocrotaline-induced renal toxicity in mice. Acta Pharmacol Sin. 35(2):292–300. 24362331 10.1038/aps.2013.145 PMC4651220

[CIT0037] Zhang XJ, He C, Tian K, Li P, Su H, Wan JB. 2015. Ginsenoside Rb1 attenuates angiotensin ii-induced abdominal aortic aneurysm through inactivation of the JNK and p38 signaling pathways. Vascul Pharmacol. 73:86–95. 25912763 10.1016/j.vph.2015.04.003

[CIT0038] Zhuang W, Lian G, Huang B, Du A, Xiao G, Gong J, Xu C, Wang H, Xie L. 2018. Pulmonary arterial hypertension induced by a novel method: twice-intraperitoneal injection of monocrotaline. Exp Biol Med (Maywood)). 243(12):995–1003. 30099957 10.1177/1535370218794128 PMC6180403

